# Facial masks in children: the position statement of the Italian pediatric society

**DOI:** 10.1186/s13052-020-00898-1

**Published:** 2020-09-15

**Authors:** Alberto Villani, Elena Bozzola, Annamaria Staiano, Rino Agostiniani, Antonio Del Vecchio, Nicola Zamperini, Francesco Marino, Davide Vecchio, Giovanni Corsello

**Affiliations:** The Italian Pediatric Society, Rome, Italy

**Keywords:** Facial mask, Children, COVID, Fake news

## Abstract

Facial masks may be one of the most cost-effective strategies to prevent the diffusion of COVID 19 infection. Nevertheless, fake news are spreading, alerting parents on dangerous side effects in children, such as hypercapnia, hypoxia, gut dysbiosis and immune system weakness. Aim of the Italian Pediatric Society statement is to face misconception towards the use of face masks and to spread scientific trustable information.

## Background

The World Health Organization (WHO) and the Centers for Disease Control and Prevention (CDC) state face masks help to prevent the spread of the coronavirus [1, 2]. In fact, masks may provide a barrier for potentially infectious droplets where physical distancing of at least one meter is not possible. According to Italian government, facial masks are required to children aged over 6 months in closed public setting and whenever social distancing measures are difficult to maintain are important. The Italian Pediatric Society suggests facial mask protection also in children over 3 years old. In case of younger children, as well as of children affected by underlying diseases not compatible with a face mask protection, personal protective equipment should be used by caregivers [3].

Nevertheless facial masks should be elastic to fit children face and made by hypoallergenic and breathable material to avoid suffocation, misleading claims about the health risks of face masks are spreading.

## The fake news and the role of the Italian pediatric society

Some people state that face masks may be dangerous and even life-threating for children. In details, they say that wearing a mask may restrict their breathing, reduce the intake of oxygen and force children to breathe their own carbon dioxide, causing hypercapnia. As a consequence, children consciousness may be affected, causing hypoxia and leaving them feeling faint, light-headed, or “smothered.” Facial mask may be unconfortable but they not have an impact on healthy children aged over 3 years. Surgeons daily wear face coverings for many hours without coming to harm.

As well as the WHO, the Italian Pediatric Society states that the prolonged use of medical masks, when properly worn, does not cause carbon dioxide intoxication nor oxygen deficiency in healthy children.

False claims about coronavirus include shuts down immune system and increases virus risk. Masks can be effective in preventing the spread of respiratory viruses such as COVID-19 from person to person, avoiding droplets land on mouth or nose [2]. Recent studies found out that surgical face masks could prevent transmission of human coronaviruses and influenza viruses from symptomatic individuals [4]. The Italian Pediatric Society provided social campaigns in order to drop out the misinformation that facial masks may trigger an infection and causes disease.

Another false claim is about no peer-reviewed studies into the effectiveness of masks within a social environment. The Lancet journal published the findings of an international research team that conducted a systematic review of 172 studies assessing distance measures, face masks and eye protection to prevent transmission of three diseases caused by coronaviruses - COVID-19, SARS and MERS [5] Mass masking for source control is in our view a useful and low-cost adjunct to social distancing and hand hygiene during the COVID-19 pandemic [6].

Finally, gut dysbiosis has been linked to facial mask use in COVID-19 pandemic. Nevertheless, no scientific evidence demonstrated an alteration of gut microbiome. Of note, the Italian Pediatric Society recommend to educate children wearing facial masks after properly washing hands and substituting them if they get dump or wet.

## Discussion

Misconceptions and misinformation towards the use of face masks may hinder the containment of the COVID-19 pandemic. The “anti-mask” sustainers alert people to potentially harmful side-effects, publishing post on internet (you tube, facebook, etc) which may be re-edited and shared several times, becoming viral. The Italian Pediatric Society addresses the fake news by analyzing the advice on the community use of masks across different credible health authorities, such as WHO and CDC, and scientific reports. The Italian Pediatric Society promotes the use of masks among the pediatric population, acknowledging that they are effective and explaining the importance of their proper use along with other hygiene measures. Mask should be worn depending on the social situation, educating both children and parents on their appropriate use.

The approach that the Italian Pediatic Society has been using to properly address the use of masks on social media focus on the connection between affordable scientific information and the deep knowledge of how social networks work and how the information spread on the Internet. The strategy is based on the following point: it is not only important to provide verified information, but it’s fundamental that any information is designed by the rules of the so called ‘algorthmic compliance’. In other words, the focus must be in designing validated pieces of content that, at the same time, follow the rules of the most important digital platforms’ algorithms [7].

At the same time, the information flow is consistent, in order to offset any kind of fake news. On the Internet – and on the social network, mostly – the most important value is users’ attention. Producing a relevant number of pieces of content and sharing them continuously give better chance to win the battle for attention.

## Conclusions

Communication and preparedness related to the usefulness of facial masks and to the lack of pediatric side effects are required to prevent infection spread. The Italian Pediatric Society statement faces misconception towards the use of face masks and highlights scientific trustable information on facial masks.

## Data Availability

Bambino Gesù Children Hospital, Dr. Bozzola’s repository.
